# Wolfram Syndrome 1: A Pediatrician’s and Pediatric Endocrinologist’s Perspective

**DOI:** 10.3390/ijms24043690

**Published:** 2023-02-12

**Authors:** Anastasios Serbis, Dimitrios Rallis, Vasileios Giapros, Assimina Galli-Tsinopoulou, Ekaterini Siomou

**Affiliations:** 1 Department of Pediatrics, School of Medicine, University of Ioannina, 451 10 Ioannina, Greece; 2 Neonatal Intensive Care Unit, School of Medicine, University of Ioannina, 451 10 Ioannina, Greece; 3 Second Department of Pediatrics, School of Medicine, Faculty of Health Sciences, AHEPA University General Hospital, Aristotle University of Thessaloniki, 541 24 Thessaloniki, Greece

**Keywords:** Wolfram syndrome 1, diabetes mellitus, diabetes insipidus, endoplasmic reticulum dysfunction

## Abstract

Wolfram syndrome 1 (WS1) is a rare autosomal recessive neurodegenerative disease caused by mutations in *WFS1* and *WFS2* genes that produce wolframin, a protein involved in endoplasmic reticulum calcium homeostasis and cellular apoptosis. Its main clinical features are diabetes insipidus (DI), early-onset non-autoimmune insulin-dependent diabetes mellitus (DM), gradual loss of vision due to optic atrophy (OA) and deafness (D), hence the acronym DIDMOAD. Several other features from different systems have been reported such as urinary tract, neurological, and psychiatric abnormalities. In addition, endocrine disorders that can appear during childhood and adolescence include primary gonadal atrophy and hypergonadotropic hypogonadism in males and menstrual cycle abnormalities in females. Further, anterior pituitary dysfunction with deficient GH and/or ACTH production have been described. Despite the lack of specific treatment for the disease and its poor life expectancy, early diagnosis and supportive care is important for timely identifying and adequately managing its progressive symptoms. The current narrative review focuses on the pathophysiology and the clinical features of the disease, with a special emphasis on its endocrine abnormalities that appear during childhood and adolescence. Further, therapeutic interventions that have been proven to be effective in the management of WS1 endocrine complications are discussed.

## 1. Introduction

Wolfram syndrome 1 (WS1) is a rare neurodegenerative disease first described in 1938 by Wolfram and Wagner in a family of eight siblings, four of whom suffered from optic nerve atrophy and juvenile diabetes mellitus [1]. More recently, the disease has been described by the acronym DIDMOAD, since its main features include diabetes insipidus (DI), early-onset non-autoimmune insulin-dependent diabetes mellitus (DM), in addition to the gradual loss of vision due to optic atrophy (OA) and deafness (D). Several other features are present in patients with WS1 including temperature regulation, urinary tract, and bowel abnormalities as well as neurological, psychiatric, and endocrinological disorders such as gonadal, adrenal, and pituitary malfunction [2].

The classical form of WS1 is transmitted in an autosomal recessive mode and is caused by mutations in the *Wolfram syndrome 1* (WFS1) gene, which is located on chromosome 4p16.1 and is responsible for the production of wolframin [3]. Wolframin is highly expressed in brain tissue, pancreatic cells, heart, lung, and placenta, and is involved in endoplasmic reticulum (ER) calcium (Ca^2+^) homeostasis and cellular apoptosis [4]. More recently, atypical forms of WS1 have been described due to various mutations of the WFS1 gene, such as a dominant and a recessive Wolfram-like disease without full-blown DM [5,6,7]. Patients with a similar disease named Wolfram syndrome 2 (WS2) share some of the WF1 clinical features, namely DM, high frequency sensorineural hearing loss, and optic atrophy. WS2 is caused by autosomal recessive mutations in a different gene, the *CISD2* (CDGSH iron sulfur domain 2) gene which is located on chromosome 4q22-q23 [8]. In addition, patients with WS2 do not present DI or psychiatric disorders, and they are characterized by defective platelet aggregation, bleeding, and peptic ulcers [9]. Since WS2 is genetically and clinically a distinct entity, it will not be dealt with in this review.

WS1 is a rare disease with a prevalence of 1/770,000 in the general population and 1/500,000 in the pediatric population (from a study in the UK) [2,10]. A similar prevalence has been described in the general population in Japan (1/710,000) [11], but is considered to be more common in North America (1/100,000) [12], and even more so in areas with high rates of consanguinity, such as Lebanon (1/68,000) [13] and a district in North-Eastern Sicily (1/54,478) [14]. Since it is a rather rare disease presenting initially with insulin-dependent DM in childhood, WS1 is frequently misdiagnosed, at least at its onset. Indeed, WS1 diagnosis delay was at least 7 years in a study from Poland, where all patients in a pediatric cohort were initially diagnosed with type 1 DM with ophthalmic complications due to diabetes [15]. The prevalence of WS1 among patients with DM ranges between 0.57% in the UK [16] to 4.8% in Lebanon [13], and has been shown to be 26 to 35 times less frequent than monogenic diabetes (Maturity onset diabetes of the young -MODY- and neonatal diabetes) in the Polish pediatric population [17].

Early diagnosis of the disease is essential in order to adequately follow patients with WS1, to timely identify its progressive and debilitating symptoms and to offer the much-needed supportive care. In addition, genetic counseling is essential for the patient’s family [2]. Unfortunately, prognosis for patients with WS1 is poor, as the disease is rapidly progressive, leading to death usually by the fourth decade of life, mainly due to brainstem atrophy and respiratory failure [18]. Currently, no cure is available for the disease, but as its pathophysiology is gradually revealed, more specialized treatment options could be developed [19].

The aim of the current narrative review is to focus on the pathophysiology and clinical features of the disease, with a special emphasis on its early appearing endocrine abnormalities. Further, possible therapeutic interventions are discussed that could improve patients’ quality of life, decelerate the disease progression, and ultimately increase their life expectancy.

## 2. Methods

A literature search on PubMed database was conducted up to 30 September 2022, to identify relevant papers using the following keywords: “Wolfram syndrome 1, WS1, pediatric, child, adolescent, endocrine, treatment”. Limitations included articles in English and patients aged 0–18 years. Clinical case reports, clinical case series, observational studies, and reviews were all included in the initial evaluation. Duplicates and relevance were initially evaluated according to title and abstract, where available. Full-text articles for all relevant studies were retrieved and reviewed. Additional relevant papers that were identified through a manual search of the references from the retrieved articles, were also included.

## 3. Results

The initial literature search identified 285 articles of which, 62 were deemed irrelevant based on their titles and 104 based on their abstract. In addition, 16 articles were not in the English language. Of the 103 full-text articles that were retrieved and reviewed, 82 were considered pertinent for this review. In addition, 12 more articles were identified as pertinent, after manual search of the references of the retrieved articles. In the end, 94 articles were included in the current review. Due to the rarity of the disease and the diverse populations and types of interventions described, the results are presented in a narrative, rather than in a systematic way.

## 4. Discussion

A. Genetic background and molecular pathophysiology

Inoue et al. identified in 1998 the WFS1 gene as responsible for WS1 pathogenesis [3]. WFS1 is located on chromosome 4p16.1, consists of eight exons and is responsible for the production of the protein wolframin. Wolframin is a transmembrane glycoprotein of 890 amino acids located in the ER and consists of nine transmembrane segments and a large hydrophilic region at both ends. So far, more than 200 mutations of WFS1 gene have been described, most of which are in exon 8 and most being nonsense or frameshift mutations [20]. Since exon 8 codes for wolframin transmembrane and C-terminal domain, both of which are important for its functionality, mutations in exon 8 are usually inactivating mutations [3,21]. Besides the typical WS1, atypical forms of the disease have been described due to various mutations of the WFS1 gene, such as a dominant and a recessive Wolfram-like disease without full-blown DM [5,6,7]. In addition, autosomal dominant WFS1 mutations have been implicated in non-syndromic low-frequency hearing loss with childhood onset and without any of the other WS1 manifestations [22].

Regarding WS1 pathophysiology, ER membrane malfunction seems to be the key player. ER is the largest organelle in the cell and is well known to play a major role in the process of protein synthesis, folding and transport as well as in calcium storage, carbohydrate metabolism and lipid and steroid synthesis [23]. In WS1, wolframin’s malfunction due to WFS1 mutations leads to an accumulation of misfolded proteins causing ER stress. To compensate for this increased stress and to restore its homeostasis, ER initiates the so-called unfolded protein response (UPR) that comprises transcriptional and translational modifications. The UPR ability to compensate for increased stress is finite, and in cases of chronic persistent stress, such as in some types of diabetes, in inflammatory and viral diseases, in cancer, and in WS1, it induces cell apoptosis through a process called the terminal UPR [24,25].

More specifically, ER has three transmembrane proteins that are responsible for sensing stress and for initiating cell apoptosis when needed. The first of these proteins is called inositol-requiring protein 1 (IRE1). Under physiologically increased ER stress, IRE1 activates a transcription factor called X-binding protein 1 (XBP-1), that translocates to the nucleus and upregulates certain UPR genes in order to restore homeostasis and protect the cell. In case of pathologically increased and/or chronic ER stress, IRE1 activates genes that lead to cell apoptosis. In addition, IRE1 has been shown to play a role in the biosynthesis of pro-insulin, since it is activated by transient exposure to high glucose levels and has a beneficial effect on the β-cells of the pancreas. In contrast, chronically elevated glucose levels increase ER stress and lead to IRE1 hyperactivation and suppression of the insulin gene [26].

The second ER protein that plays a role in UPR is the so-called protein kinase RNA (PKR) -like ER kinase (PERK). Under increased ER stress, this transmembrane protein activates the phosphorylation of several translation and transcription factors (such as eIF2alfa and ATF4) which on one hand decrease ER protein biosynthesis, and on the other, facilitate amino acid transport and metabolism and activate antioxidant mechanisms. In case of protracted pathological stress, the activation of PERK can lead to cell apoptosis through alternative gene activation [19].

Activating transcription factor 6 (ATF6) is the third transmembrane protein involved in UPR. In case of increased stress, ATF6 translocates to the Golgi apparatus where it is activated to an active transcription factor. Subsequently, it moves to the nucleus where it upregulates genes that improve protein folding, processing, and degradation in the ER of the cell, thus restoring homeostasis [27,28,29]. In WS1, hyperactivation of ATF6 leads to cell apoptosis by upregulating the corresponding genes. Further to the above-mentioned proteins, ER chaperones such as immunoglobulin binding protein (BIP), while being inactive under physiological conditions, in case of high stress levels they are recruited in the ER lumen to facilitate the folding of accumulated proteins [30]. In addition, recruited BIP dissociate from IRE1, PERK, and ATF6, leading to their activation and the initiation of the UPR. To safeguard a balance between hypo- and hyperactivation of the stress-induced UPR in the ER, the WFS1 gene acts as a negative regulator that, under physiological ER stress, inhibits the activation of ATF6 and suppresses stress signals.

Several other mechanisms have been suggested to be implicated in WS1 pathogenesis. For example, since wolframin plays an important role in ER Ca^2+^ homeostasis and Ca^2+^ signal transduction processes involved in apoptosis, imbalances in cytosolic Ca^2+^ levels that activate Ca^2+^-dependent proteases and lead to apoptosis have been described in WS1 patients [31,32,33]. In addition, mitochondrial dysfunction and mitochondria-associated ER membranes (MAMs) damage have been identified in WS1 patients, which may also play an important role in pathogenesis of the disease [29,34]. Indeed, a recent study showed that ER stress, impaired Ca^2+^ homeostasis, altered mitochondrial function, and delayed neuronal development are causatively related events [35].

B. Clinical features

**Figure 1:**
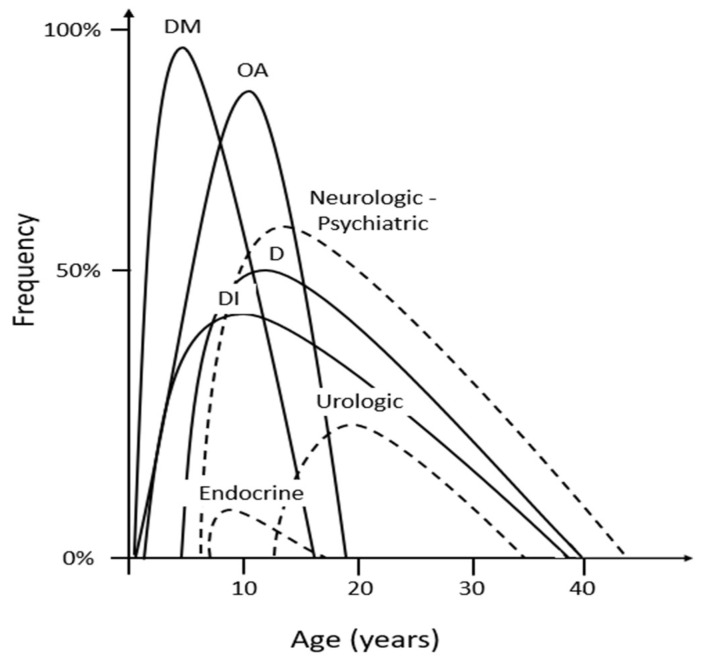
Prevalence of various features of WS1 (main features with continuous and secondary features with dashed line) and the age at which these features are diagnosed according to data from reference [36]. D: deafness, DI: diabetes insipidus, DM: diabetes mellitus, OA: optic atrophy.

Despite the recently described atypical forms of WS1, the classical features of the typical autosomal recessive WS1 are DM, central DI, OA and sensorineural hearing loss (Table 1). Several other features of the disease have been described in various cohorts of patients either from the endocrine or -more frequently-, from the nervous, urinary, and other systems [2,36]. The exact frequency with which these symptoms appear, the order of appearance, as well as the age at which they manifest differ between patients. Further, despite several efforts, no clear correlation between genotype and phenotype has been established, partly because of the complexity of the gene and because of the rarity of the disease [37,38,39]. Several cohorts of WS1 patients were used to establish the prevalence of its various features and the age at which these features are diagnosed [13,16,36,40,41,42]. In Table 1 and in Figure 1, the frequency of each of the manifestations of the disease is described according to the largest to date such cohort that included 412 patients with WS1 [36].

In the following paragraphs, the main endocrine and non-endocrine manifestations of WS1 are described in more detail, followed by the description of less frequent endocrine and non-endocrine manifestations.

## 5. Main Clinical Features

### 5.1. Diabetes Mellitus

DM is usually the first manifestation of the disease diagnosed in childhood, usually around the early school age (6 years) with a range between 3 weeks to 16 years. This diabetes is a non-autoimmune, usually non-HLA-linked [43], insulin-dependent diabetes that can be easily misdiagnosed at its onset as T1DM. As already discussed in the Introduction section, studies have shown that diagnosis of WS1 can be delayed for several years because children with the disease are initially misdiagnosed with T1DM [15].

Regarding the pathomechanism of WS1 diabetes, it is known that the pancreatic β-cells highly express WFS1 gene. Since wolframin is malfunctioning in WS1 patients, there is a chronic accumulation of misfolded proteins in their β-cells ER leading to stress-induced inflammation and ultimate apoptosis [44]. In addition, WFS1 gene has been shown to be upregulated in β-cells during insulin secretion, suggesting a possible paramount role in proinsulin folding and processing in their ER [45,46]. The gradual malfunction of the β-cells and the reduction in their number due to apoptotic death, lead to a decrease in insulin production and thus to hyperglycemia and diabetes [25]. Over time, β-cell malfunction could be further accentuated by the progressive hyperglycemia, since it has been shown that chronic exposure to high glucose levels causes ER stress and hyperactivation of both IRE1 and ATF5, leading to the suppression of insulin gene [26].

Nevertheless, it seems that the decline in insulin levels is more gradual in WS1 diabetes compared to T1DM. Therefore, patients with WS1 appear much less frequently with ketoacidosis, have lower insulin requirements, smaller glycemic variability, lower HbA1c levels, and a longer remission period [47,48]. In addition, a smaller frequency of microvascular complications in these patients could be attributed to a longer persistence of residual insulin secretion [47]. On the contrary, hypoglycemia episodes seem to be more frequent in patients with WS1, possibly due to the dysfunction of the autonomic nervous system caused by a perturbation of ER function [48]. Rarely, co-existence of WS1 and T1DM has been described in patients diagnosed with diabetes and characterized by positive insulin antibodies, together with other typical features of WS1 [49]. Furthermore, recent studies have described patients with WS1 and type 2 diabetes (T2DM), suggesting a possible link between WFS1 gene, among other genes, with T2DM [50,51]. The differences between WS1 diabetes and T1DM are shown in Table 2.

### 5.2. Diabetes Insipidus (DI)

Central diabetes insipidus (recently renamed to arginine vasopressin deficiency syndrome) is the other frequently encountered endocrinological abnormality in patients with WS1. Up to two thirds of the patients present DI at an average age of 14 years (3 months to 40 years). In many cases DI is partial and, therefore, quite difficult to diagnose, at least initially [36]. A high index of suspicion is required and patients with diabetes, vision and hearing defects, neurological abnormalities or any other feature of WS1 should be tested with a water deprivation test in order to timely establish the diagnosis. Treatment of DI in WS1 patients is with intranasal or oral desmopressin, to which most of the patients respond well.

### 5.3. Optic Atrophy

Loss of vision due to OA is one of the four criteria required for the diagnosis of WS1 and usually appears in early adolescence (average age 11 years) with a wide range of 6 weeks to 19 years [36]. The ophthalmologic findings include cataracts, nystagmus, abnormal pupillary light reflexes, and glaucoma that gradually lead to a progressively decreasing visual acuity and color vision defects [18,52]. In addition, retinal pigmentary changes, such as pigmentary maculopathy, have rarely been described in patients with WS1 [53]. Recent studies have shown that the retinal thickness, measured with high-definition optical coherence tomography (OCT) and/or magnetic resonance imaging (MRI), is lower in subjects with WS1 compared to patients with T1DM or healthy controls, and that high-definition OCT could be a valuable tool for adequate monitoring of patients with WS1 over time [54,55].

### 5.4. Deafness

A progressive hearing loss is characteristic of WS1 patients. It usually presents during the second decade of life, at an average age of 12.5 years (range 5–39 years) and it is seen in more than half of the patients with the disease (62%) [10,36]. Some but not all of the studies show a faster hearing deterioration in females compared to males [10,56]. High frequencies seem to be affected first and a gradual hearing impairment is observed, probably due to the progressive nervous system deterioration [56]. Hearing loss is usually asymptomatic and is usually identified on audiometric testing [41]. Therefore, patients with WS1 must be followed up yearly or every other year with an audiometry test and an auditory brainstem response for early identification of any hearing deterioration and for timely intervention with hearing aids and cochlear implants [57].

## 6. Less Frequent Non-Endocrine Features

### 6.1. Neurological and Autonomic Complications

According to the study by De Heredia et al. [36]. neurological and autonomic disorders appear at an average age of 16 years (5–44 years) and are diagnosed in almost one fifth of WS1 patients (Table 1). However, other studies have shown a much higher prevalence of neurological abnormalities reaching up to 70% [14,16] and with a much earlier onset [42]. Amongst these abnormalities, cerebellar ataxia is the most common, with dysarthria, dysphagia, epilepsy, areflexia, myoclonus, and progressive brainstem atrophy being less frequently encountered [16]. Neurological complications are important causes of morbidity and mortality, since dysphagia and loss of gag reflex can lead to aspiration pneumonia and brainstem atrophy to central apnea, a major cause of death in these patients. Polysomnography and overnight oximetry tests can help in the follow-up of these patients [10,16]. Regarding autonomic nervous system dysfunction, WS1 patients may present with sweating problems (anhidrosis, hypo- or hyperhidrosis), orthostatic hypotension, disorders of thermoregulation (hypo- or hyperthermia), gastrointestinal motility abnormalities (gastroparesis, constipation, stool incontinence), and headaches [21,36,42].

### 6.2. Psychiatric Abnormalities

Several psychiatric disorders have been described in WS1 patients that usually appear by early adulthood (average age 20.5 years) in almost half of the patients [36]. More specifically, anxiety, panic attacks, mood swings, sleep abnormalities, psychosis, impulsive behavior and aggressivity, as well as severe depression have been reported [16,36,58]. WS1 patients with suicide attempts have been described making the multidisciplinary management and follow-up of these patients imperative [59,60].

### 6.3. Urological Abnormalities

Urological abnormalities are frequently reported in WS1 patients, usually due to autonomic dysfunction leading to neurogenic bladder and urinary incontinence, upper tract dilatation and recurrent infections. The prevalence of these disorders varies greatly in various studies ranging between 19 and 90%, with a median age of onset around early adulthood (20 years of age) [36,61]. Rarely, WS1 patients have been reported to progress to chronic renal failure requiring hemodialysis [61,62]. Therapy for urinary bladder dysfunction consists of anticholinergic agents and intermittent catheterization.

## 7. Less Frequent Endocrine Features

Several male patients with WS1 have been reported with both hypergonadotropic (peripheral) and hypogonadotropic (central) hypogonadism, even if this finding is not frequently reported [13,16,63,64]. Hypogonadism is usually diagnosed during puberty with delayed maturation of secondary sexual characteristics, small testes and penis and lack of virilization. In female patients, ovarian function seems to be intact and only menstrual cycle abnormalities have been described [13,65]. The reason for this selective gonadal axis involvement in males with WS1 is not well understood. Very recently, a case of an adolescent girl with hypergonadotropic hypogonadism has been described [66].

Two other endocrine axes have been reported to be affected in patients with WS1. Firstly, deficient growth hormone (GH) production has only rarely been described, even if short stature is commonly observed in patients with WS1 [67]. In a study from Lebanon by Medlei et al. the most commonly identified abnormality in pituitary function was deficient GH secretion, which was found in almost half of the patients that were examined (9 out of 20) [13]. It is therefore important to appropriately follow up WS1 patients with at least yearly height measurements and GH stimulation tests if needed, in order to timely identify height gain deceleration that could respond to rhGH administration [10].

Anterior pituitary dysfunction has been associated with deficient ACTH secretion as well. In the above-mentioned study from Lebanon, 4/20 WS1 patients examined, exhibited defective cortisone secretion, most probably of central etiology [13]. Since similar findings have been reported from other studies as well, the administration of cortisone at times of increased stress, e.g., during a severe infection, should be considered in such patients [10,13,18]. In addition, careful adjustment of the insulin dosage is required in these patients during steroid substitution therapy [13].

C. Diagnostic approach

As already described, one of the main difficulties in diagnosing WS1 is that it can initially present as insulin dependent, antibody-negative diabetes mellitus. This misdiagnosis can be further complicated by the identification of vision abnormalities within 5–10 years from the diabetes diagnosis, that could be interpreted as diabetic retinopathy. Indeed, Zmylowska et al. in a recent study from Poland, observed a 7-year delay between diagnosis of OA and referral for genetic analysis [15]. A high index of suspicion for patients with early onset insulin dependent diabetes and optic atrophy before the age of 16 can help appropriately and timely diagnose the disease based on the differences between WS1 diabetes and T1DM, as already described.

The diagnosis will be confirmed by genetic testing. Even if there is still no direct genotype–phenotype correlation for WS1, early and timely diagnosis is very important both for the patient himself and his or her family. For the patient, regular follow-up, including neurological, ophthalmologic, ENT, endocrinological, and psychiatric consultations, will delay complications and will offer the best available treatments. For the patient’s family, a careful assessment of possible siblings together with a meticulous genetic counseling is recommended.

D. Treatment modalities for youth with WS1

Currently, the goals of WS1 treatment are to effectively deal with the main features of the disease as efficiently as possible and to decelerate the disease progression. Regarding WS1 diabetes, several hypoglycemic drugs that have been used for adults with T2DM, have been tried in WS1 adult patients with promising results [68]. Such drug categories include thiazolidinediones (e.g., pioglitazone) which inhibit inositol triphosphate (IP3R)-mediated release of Ca^2+^ from the ER [18], Dipeptidil Peptidase 4 inhibitors (DPP4) (e.g., sitagliptin and vildagliptin) that block the destruction of Glucagon-Like Peptide-1 (GLP-1) concentration [69], and exenatide (an incretin mimetic drug) that directly increases GLP-1 concentration. At the time, none of these drugs is approved for use in children and adolescents younger than 18 years. Nevertheless, a recent study using linagliptin (a DPP4 inhibitor) for the treatment of an adolescent patient with WS1 diabetes in addition to intensive insulin treatment, showed a dramatic decrease in exogenous insulin need with a good safety profile [70].

Two drugs that belong to the incretin mimetic group (GLP-1 analogs) have been recently approved for use in adolescents with T2DM. More specifically, liraglutide is administered subcutaneously once a day and has been shown to be efficacious in improving glycemic control over 52 weeks in youth with T2DM, albeit with the increased frequency of gastrointestinal adverse events [71]. GLP-1 analogs have been shown to play a role in decreasing ER stress-induced β-cell apoptosis and in increasing β-cell growth and survival. In addition, they seem to have a neuroprotective effect [72]. Very recently, liraglutide was shown to have good safety, tolerability, and efficacy in four patients aged between 10 and 14 years with WS1 diabetes who were treated for 8–27 months [73]. Further relevant studies are needed. Until then, a multiple daily insulin injection scheme is considered the first choice in youth with WS1 diabetes. The use of a subcutaneous insulin pump can also be considered an alternative.

A better alternative to treating WS1 patients with diabetes would be the prevention of their β-cell destruction. As described earlier, β-cell death could be partly caused by ER Ca^2+^ depletion. Thus, treatment with agents that can stabilize ER Ca^2+^ levels could prevent cell apoptosis. Dantrolene is a skeletal muscle relaxant, traditionally used in the treatment of malignant hyperthermia [74]. Dantrolene seems to block ryanodine receptors (RyRs) on the ER, thus reducing cytosolic calcium efflux and preserving ER integrity and therefore preventing β- and neural cell apoptosis. Despite some promising previous studies, a recent well-designed clinical trial failed to show any improvement in β cell function, visual acuity or neurological function of adult and pediatric WS1 patients after a 6-month dantrolene sodium therapy [75].

Carbachol, a muscarinic receptor 2 agonist which potentiates glucose-stimulated insulin secretion, could be of use in patients with WS1 diabetes. To the best of our knowledge, the only study that has tested this therapeutic approach thus far is the one by Toots et al. in mice, which showed improved insulin secretion after carbachol administration [76]. Further studies on humans, and particularly in youth, are needed.

Sodium valproate, a well-known anti-epileptic drug, is also being examined as a repurposed drug treatment for both pediatric and adult patients with WS1. This drug has been shown both in vitro and in vivo studies to have a neuroprotective effect by inhibiting ER stress-induced apoptosis [77,78]. Therefore, a phase II randomized, double-blind, placebo-controlled trial in WS1 patients aged ≥5 years is in progress at the University of Birmingham, UK (ClinicalTrials.gov: NCT03717909) [79]. The treatment options that have been tried in patients with WS1 diabetes are collectively described in Table 3.

More advanced treatment modalities mainly for adult patients with WS1, such as the use of chemical chaperones to assist ER protein folding, the transplantation of appropriately managed pluripotent stem cells to regenerate affected tissues, or gene therapy by using viruses or the CRISPR technology, are being investigated in specialized centers around the world [81]. Hopefully, these therapeutic interventions will be available to WS1 patients in the near future, so as to improve their quality of life and to decelerate or even stop the progression of the disease, with the ultimate goal of increasing their life expectancy.

## 8. Concluding Remarks

WS1 is a rare progressive disease, with signs and symptoms originating from several different systems and organs that usually start appearing in childhood. Due to rarity of the disease and the sequential involvement of the different systems, its diagnosis is usually delayed by several years. A high index of suspicion is required in pediatric patients with non-autoimmune insulin-dependent diabetes that is accompanied by vision loss, hearing abnormalities, diabetes insipidus or any of the other endocrine and non-endocrine features of WS1. The recent progress in the understanding of the disease pathophysiology, the availability of a rapidly increasing armamentarium of drugs that could help in reducing morbidity and mortality, and the future perspectives of gene therapy and tissue regeneration increase the possibilities for better management of both pediatric and adult patients with WS1 in the near future. Until then, timely diagnosis and appropriate symptomatic and palliative treatment can make the difference in the quality of life of these patients.

## Figures and Tables

**Table 1:** Major and secondary features of Wolfram syndrome 1 [36].

Clinical Features	Average Age at Diagnosis (Range)	Percentage of Patients with the Specific Feature
Major endocrine features		
Diabetes mellitus	6 years (3 weeks–16 years)	98.2%
Central diabetes insipidus	14 years (3 months–40 years)	37.7%
Major non-endocrine features		
Optic atrophy	11 years (6 weeks–19 years)	82.1%
Sensorineural hearing loss	12.5 years (5–39 years)	48.2%
Secondary endocrine features	8 years (7–9 years)	6.6%
Hypogonadism	Puberty	5%
Delayed menarche in females	Puberty	<1%
Deficient GH secretion	Childhood	3%
Corticotropin deficiency	Childhood	1.3%
Secondary non-endocrine features		
Neurological and autonomic disorders	16 years (5–44 years)	17.1%
Psychiatric symptoms	20.5 years (17–23 years)	44.4%
Urinary tract complications	20 years (13–33 years)	19.4%

**Table 2:** Differences between Wolfram syndrome 1 diabetes and type 1 diabetes mellitus [18,43,47,48,49].

Characteristic	Wolfram Syndrome Diabetes	Type 1 Diabetes Mellitus
Ketoacidosis at presentation	3%	Up to 1/3 of patients
Presence of other clinical features of WS1	Yes	No
HLA subtype	Almost half with HLA-DR2	Mainly HLA-DR3 and HLA-DR4
Presence of insulin autoantibodies	Very rarely (coexistence with T1DM?)	>90%
Insulin requirement	Lower doses	Large basal and bolus doses
Remission (honeymoon) period	Longer	Weeks to months
Diabetic retinopathy	35%	90%
Diabetic nephropathy	8%	27%
Median age of death	30–40 years	60–70 years
Cause of death	Neurological disorder, urological abnormalities	Macrovascular complications (myocardial infarction, stroke)
Next pregnancy recurrence risk	25% (autosomal recessive)	3–6%

**Table 3:** Drugs used to treat diabetes in young patients with Wolfram syndrome 1.

Compound	Mechanism of Action	Status of Use	References
Subcutaneous Insulin	Substitutes pancreatic insulin	Main therapy option in children with WS1 diabetes	[80]
Liraglutide	Glucagon-like peptide-1 (GLP-1) receptor agonist. It increases cyclic AMP stimulating the glucose dependent release of insulin and interferes with the ER unfolded protein response	Recently approved for use in children >12 years with type 2 diabetes. A single study in children with WS1 diabetes	[71,73]
Linagliptin	A Dipeptidyl Peptidase 4 (DPP-4) inhibitor, an enzyme that degrades the incretin hormones GLP-1 and glucose-dependent insulinotropic polypeptide (GIP).	Not approved for patients <18 years. A single study on its use (added to insulin regimen) in an adolescent with WS1 diabetes	[70]
Pioglitazone	Inhibits inositol triphosphate (IP3R)-mediated release of Ca^2+^ from the ER	Not approved for patients <18 years. Has been tried only in adults with WS1 diabetes	[18]
Dantrolene	A skeletal muscle relaxant, seems to block ryanodine receptors on the ER, reducing cytosolic calcium efflux and preserving ER integrity thus preventing β-cell apoptosis	Clinical trial with adult and pediatric WS1 patients receiving dantrolene for 6 months	[75]
Carbachol	A muscarinic receptor 2 agonist which potentiates glucose-stimulated insulin secretion	A single study in mice showed improved insulin secretion after carbachol administration	[76]
Sodium valproate	Considered to increase production of protein p21, which prevents cell apoptosis. It is also thought to increase wolframin production	A phase II randomized, double-blind, placebo-controlled trial in WS1 patients aged ≥5 years is in progress	[79]

## Data Availability

Not applicable.
